# Depersonalization Disorder: Disconnection of Cognitive Evaluation from Autonomic Responses to Emotional Stimuli

**DOI:** 10.1371/journal.pone.0074331

**Published:** 2013-09-13

**Authors:** Matthias Michal, Ansgar Koechel, Marco Canterino, Julia Adler, Iris Reiner, Gerhard Vossel, Manfred E. Beutel, Matthias Gamer

**Affiliations:** 1 Department of Psychosomatic Medicine and Psychotherapy, University Medical Center, Mainz, Germany; 2 Medical Psychology and Medical Sociology, University Medical Center, Mainz, Germany; 3 Institute of Psychology, Department of General & Experimental Psychology, Johannes Gutenberg-University, Mainz, Germany; 4 Department of Systems Neuroscience, University Medical Center Hamburg-Eppendorf, Hamburg, Germany; University of Medicine & Dentistry of NJ - New Jersey Medical School, United States of America

## Abstract

**Background:**

Patients with depersonalization disorder (DPD) typically complain about emotional detachment. Previous studies found reduced autonomic responsiveness to emotional stimuli for DPD patients as compared to patients with anxiety disorders. We aimed to investigate autonomic responsiveness to emotional auditory stimuli of DPD patients as compared to patient controls. Furthermore, we examined the modulatory effect of mindful breathing on these responses as well as on depersonalization intensity.

**Methods:**

22 DPD patients and 15 patient controls balanced for severity of depression and anxiety, age, sex and education, were compared regarding 1) electrodermal and heart rate data during a resting period, and 2) autonomic responses and cognitive appraisal of standardized acoustic affective stimuli in two conditions (normal listening and mindful breathing).

**Results:**

DPD patients rated the emotional sounds as significantly more neutral as compared to patient controls and standardized norm ratings. At the same time, however, they responded more strongly to acoustic emotional stimuli and their electrodermal response pattern was more modulated by valence and arousal as compared to patient controls. Mindful breathing reduced severity of depersonalization in DPD patients and increased the arousal modulation of electrodermal responses in the whole sample. Finally, DPD patients showed an increased electrodermal lability in the rest period as compared to patient controls.

**Conclusions:**

These findings demonstrated that the cognitive evaluation of emotional sounds in DPD patients is disconnected from their autonomic responses to those emotional stimuli. The increased electrodermal lability in DPD may reflect increased introversion and cognitive control of emotional impulses. The findings have important psychotherapeutic implications.

## Introduction

Depersonalization disorder (DPD) is characterized by persistent or recurrent depersonalization, i.e. experiences of unreality, detachment, or being an outside observer with respect to one’s thoughts, sensations, actions or feelings and is often accompanied by derealization, i.e. experiences of unreality or detachment with respect to surroundings. During these experiences reality testing remains intact, and these symptoms are not caused by direct physiological effects (e.g. drugs, seizures) or better explained by another mental disorder (e.g. panic disorder). Finally, these symptoms are a source of significant burden or impairment (DSM-V) [1,2]. The prevalence of DPD is around 1% in the general population, DPD has a high comorbidity with depression and anxiety disorders, and its course is typically chronic [2-7]. Depersonalization (DP) is considered as a hard-wired stress response in reaction to extreme anxiety comprising increased alertness and suppression of emotions by prefrontal inhibition [8,9]. From a psychodynamic perspective, DPD constitutes a mental escape from experiencing anything fully by suppressing emotional experiencing [10,11]. DPD patients become detached observers of themselves and their surroundings [12-14]. This detachment affects all aspects of experience, e.g. emotion processing, thinking and body experiencing [13]. Thus, DPD sufferers typically complain of emotional numbness, i.e. that they feel nothing anymore, while at the same time, their psychomotor expression of emotions appear normal [15,16]. Recently, one neuroimaging and two psychophysiological studies found reduced autonomic responsiveness to unpleasant emotional stimuli and reduced limbic activation as compared to patients with anxiety disorders [17-19]. These findings support the cortico-limbic disconnection model of DPD, postulating that prefrontal inhibition of limbic areas, presumably mediated via attentional mechanisms, impairs “emotional coloring” of perceptions and cognitions [9]. Another recent psychophysiological study stimulated DPD patients and healthy controls with a frightening video clip over a longer duration [20]. While maximum skin conductance levels were not different between the DPD patients and healthy persons, DPD patients had higher resting baseline skin conductance levels and showed no recovery of their skin conductance level after clip offset. Likewise, it was reported that patients with DPD had higher resting baseline skin conductance levels as compared to healthy controls [21].

The antithesis of depersonalization is mindfulness, i.e. nonjudgmental attention to present-moment experiences [11,14]. A robust and specific inverse correlation of dispositional mindfulness with severity of depersonalization has been demonstrated [11]. Mindfulness-based interventions have shown promising effects for various mental disorders (e.g. major depression, anxiety disorders, and personality disorders), chronic medical conditions and for stress-reduction in healthy subjects [22-24]. Improvement of affect regulation is considered as the therapeutic mechanism of mindfulness exercises [25]. It is supposed that mindfulness promotes tolerance of negative affects and improved awareness for the body by directing attentional resources towards a limbic pathway for present-moment sensory awareness [25]. Based on clinical experience, mindfulness interventions are regarded helpful for reducing intensity of DP and increasing emotional awareness [14,26,27], although no clinical trials have tested mindfulness based interventions for DPD patients until now.

With these findings and considerations in mind we designed the present study to investigate the autonomic responsiveness to emotional stimuli and the cognitive evaluation of those stimuli in DPD patients as compared to patient controls. As most DPD patients are comorbid with depression and anxiety [2-4,28], we selected a patient control group with equal severity of depression and anxiety. The selection of this patient control group enables us to test whether depersonalization specifically effects emotion processing beyond depression and anxiety. Differences between DPD patients and patient controls would argue against the prevailing view that depersonalization is just a negligible variant of depression and anxiety [7,8,29]. As mindfulness exercises are supposed to be helpful for DPD patients, we wanted to investigate for the first time the immediate effects of mindful breathing on autonomic responses to emotional stimuli and on severity of state depersonalization. Therefore, we analyzed autonomic responsiveness to emotional stimuli in two conditions, i.e. a normal and a mindful breathing condition. As closing eyes facilitates performance of mindfulness exercises, we selected auditory instead of visual emotional stimuli [17,30].

We hypothesized firstly, that DPD patients as compared to patient controls will show blunted autonomic responses to emotional stimuli, and secondly, that mindful breathing will increase their autonomic responsiveness and reduce depersonalization severity.

## Materials and Methods

### Participants

The study was approved by the Ethics Committee of the Rhineland-Palatinate State Board of Physicians (Germany). All participants provided their written informed consent to participate in this study. The sample consists of n=22 DPD patients and n=15 patient controls (Table 1). The diagnosis of DPD was established by M.M. according the German version of the Structured Clinical Interview for Dissociative Disorders [31]. Participants fulfilled the criteria of DPD according to DSM-IV (300.6) as well as the criteria of the depersonalization-derealization-syndrome according to ICD-10 (F48.1). Patients were recruited from the DPD clinic of the Department of Psychosomatic Medicine and Psychotherapy (Mainz, Germany). All DPD patients experienced persistent depersonalization. Persons with a lifetime diagnosis of a psychotic disorder or brain damage were not eligible. The mean duration of DPD was 11.7 years (standard deviation (SD)=7.5 years) with a range from 1-24.5 years. Current mental disorders other than DPD and psychotropic medication were comparable between the groups (see Table S1, supplemental material).

**Table 1:** Characteristics of the participants.

	DPD	Patient controls	Test^2^
	n = 22	n = 15	p
Age (years)	29.7 ± 8.5	28.7 ± 6.4	0.68
Men	45.5% (n = 10)	40.0% (n = 6)	0.74
Years of education^1^	11.4 ± 1.7	10.8 ± 1.7	0.28
CDS trait	181.8 ± 62.4	27.0 ± 23.1	<0.0001
CDS state	1094.5 ± 479.1	60.7 ± 64.5	<0.0001
DES	31.8 ± 12.4	10.9 ± 9.2	<0.0001
DES amnesia	10.6 ± 10.4	6.4 ± 8.9	0.22
DES depersonalization	55.0 ± 17.6	3.2 ± 4.2	<0.0001
BDI-II	31.8 ± 12.4	28.0 ± 12.5	0.37
BDI-II ≥ 30	50.0% (n = 11)	40.0% (n = 6)	0.55
STAI-T (trait)	60.1 ± 9.3	61.0 ± 10.7	0.77
STAI-S (state)	52.6 ± 10.5	48.4 ± 12.5	0.33
MAAS	3.1 ± 1.2	4.3 ± 1.1	0.005
CTQ total	58.7 ± 22.3	56.2 ± 25.2	0.76

Note: Data are presented as mean ± standard deviation or percentage (%) and numbers (n; CDS, Cambridge Depersonalization Scale; DES, Dissociative Experiences Scale (amnesia and depersonalization subscale); STAI, State-Trait Anxiety Inventory; BDI-II, Beck Depression Inventory; MAAS, Mindful Attention Awareness Scale; CTQ, Childhood Trauma Questionnaire

1) Years of education (without university or professional education 2) t-Test for continuous variables, chi-square test for categorical variables

### Questionnaires

The Cambridge Depersonalization Scale (CDS) [32,33] consists of 29 items and measures frequency and duration of depersonalization over the last 6 months. The state version of the CDS (S-CDS) comprises 22 items and reflects intensity of depersonalization right now. The Dissociative Experiences Scale (DES) [34] is a 28-item self-report scale that asks respondents to indicate the frequency of dissociative experiences on a 11-point-Likert-scale from 0%-100%. The scale contains several subscales. For this study we report the scores for the total scale, and the subscales amnesia and depersonalization. Dispositional mindfulness was assessed by the Mindful Attention Awareness Scale (MAAS) [35]. The Childhood Trauma Questionnaire (CTQ) [36] is a self-report scale of childhood interpersonal trauma. The Beck Depression Inventory-II (BDI-II) [37] is a 21-item inventory that measures depression. The State-Trait Anxiety Inventory intends to tap trait and state anxiety (STAI-T/-S) [38]. The trait questionnaires were administered within 3 days prior to the experiment (further information about the questionnaires is given in the supplemental material, Text S1).

### Instruments

Physiological responses were recorded with a Biopac MP100 system (Biopac Systems, Inc.) and stored at 200 Hz for further offline processing. Skin conductance was measured by a constant voltage system (0.5 V) using a bipolar recording with two Ag/AgCl electrodes (0.8 cm diameter) filled with 0.05 M NaCl electrolyte. The electrodes were placed at the palmar surface of the medial phalanx of the index and second finger of the non-dominant hand. An electrocardiogram (ECG) was measured with pre-gelled electrodes placed at the right arm and left leg.

### Stimulus material

A set of acoustic emotional stimuli from the International Affective Digitized Sounds (IADS) [30] was used for emotional stimulation. These sounds have a duration of 6 seconds each. They evoke scenarios of different emotional valence and arousal. Based on the normative ratings for valence and arousal [30], we selected a set of 20 sounds from the IADS: Four neutral sounds (neutral valence and low arousal: #132, #262, #720, #725) and four sets with four emotional sounds each that differed on the dimensions valence and arousal. This 2 × 2 classification included sounds with negative valence and medium arousal (#280, #319, #380, #706), negative valence and high arousal (#276, #277, #278, #279), positive valence and medium arousal (#221, #351, #602, #721); and positive valence and high arousal (#810, #815, #816, #820). Additionally, 2 low arousing positive sounds (#151, #812) were selected and used as buffers to familiarize subjects with the sound stimulation and to adjust the volume individually. These sounds were always presented at the beginning of the stimulation period and physiological responses to these sounds were not analyzed. Before running the experiment with patients, all sounds were tested in an independent sample of healthy controls to obtain normative ratings for a German sample and to examine the influence of valence and arousal on electrodermal and heart rate responses (see supplemental online material, Text S2).

### Experimental design

The experiment started with a 5 min rest period. Patients were instructed to sit quietly on a comfortable chair with their eyes closed. Electrodermal and heart rate data were acquired continuously during this rest period to allow for analyzing tonic physiological activation in DPD patients as compared to patient controls.

Subsequently, participants were informed about the upcoming stimulation period. They were asked to keep their eyes closed and listen to the sound presentation via circumaural earphones. The acoustic stimuli were presented in a random order (with the exception of the two buffer sounds that were always presented as first and second stimulus) while continuously measuring autonomic physiological responses. For presentation we used a Dell Latitude E6500 laptop with an Intel IDT 92HD71B7 HD Audio (ICH9) soundcard and AKG K141 MKII circumaural headphones. The volume was adjusted individually, so that the patients felt comfortable with the volume. Each sound of 6 seconds duration was followed by a jittered pause that lasted between 10 and 20 s (M=11.3 s, SD=1.8 s). Measurements were obtained in two conditions, whose sequential order was counterbalanced across subjects: a) Listen to the sounds and be attentive to the emotional scenarios b) breathe mindfully while listening to the sounds, i.e. direct your the attention to the bodily sensations while letting yourself sink in the sound scenarios. Participants had no formal training in mindfulness exercises.

Subjective ratings of valence and arousal for all sounds were obtained in a separate session by using the Self-Assessment Manikin [39]. The Self-Assessment Manikin measures valence and arousal on a pictorial 9-point-Likert-scale (valence: 1=extremely negative, 9=extremely positive; arousal: 1=extremely calm, 9=extremely excited). Further, participants rated on a similar, self-constructed, 9-point-Likert-scale the vividness of the imagination that was triggered by the sounds (1=not vivid at all, 9=extremely vivid). Vividness of imagination was assessed, because impaired ability to generate visual images is common among DPD patients [40]. The above ratings represent the cognitive evaluation of the affective stimuli, i.e. the evaluation of the emotional valence of the stimuli, the awareness of internal bodily and mental processes such as arousal and imagination. These ratings were not accomplished by two patients from the DPD group. Thus, corresponding analyses rely on n=20 DPD patients and n=15 patient controls.

In order to capture subjective changes induced by the mindful breathing manipulation, participants rated the degree of feeling grounded in their body on a self-constructed 21-point-Likert-scale (-10=extremely detached (no-body), 0=normal, +10=grounded very much) and the overall intensity of the sounds (-10=lowest intensity, 0=normal, +10=highest intensity). Further, participants rated present depersonalization intensity by the state CDS. The participants rated their degree of feeling grounded in their body and the severity of present depersonalization directly after each condition (normal, mindful breathing). The degree of feeling grounded was not rated by two patients from the DPD group and the overall intensity rating and S-CDS was missing from one patient of the DPD group. Thus, corresponding analyses rely on n=20 or n=21 DPD patients, respectively, and n=15 patient controls.

### Quantification of physiological responses

From the electrodermal data that were recorded during the rest period, we calculated the skin conductance level (SCL) as the average low pass filtered skin conductance signal (cutoff frequency 0.05 Hz) as well as the number of nonspecific skin conductance responses with an amplitude of at least 0.02 µS [41]. Furthermore, we quantified heart rate (HR) and heart rate variability (HRV) from the ECG recordings. To this aim, R-waves were detected in the ECG recordings and visually inspected to identify artifacts. Afterwards, interbeat-intervals (IBIs) were calculated and used to compute the mean heart rate during the rest period. Statistical parameters of HRV were calculated using the software artiifact [42]. The two time-domain measures calculated from the IBIs were the standard deviation (SDNN), and the square root of the mean squared differences between successive IBIs (RMSSD). Additionally, frequency domain measures were derived using fast Fourier transformation. According to previous recommendations [43], frequency bands were labeled as high-frequency (HF, 0.15–0.4 Hz) and low-frequency (LF, 0.04–0.15 Hz) and expressed in power (ms^2^) and normalized units (n.u.). We specifically analyzed HF and time domain measures as indicators for cardiac-vagal tone and additionally calculated LF/HF that can be interpreted as a measure of sympathovagal balance. The quality of the ECG recording of one DPD patient was insufficient for further analyses. Therefore, HR measures were analyzed for n=21 DPD patients and n=15 patient controls.

To quantify phasic physiological responses to the emotional sounds, we measured skin conductance response (SCR) amplitudes and heart rate changes. For SCR quantification, we measured the largest increase in skin conductance during a period from 1 to 8 s after stimulus onset as change in μS. These values were log-transformed to reduce the skew of the amplitude distribution [44]. For the analysis of heart rate responses, R-waves were detected in the ECG data. The automatically detected R-waves were visually inspected and R-R intervals were converted to HR (in beats per minute). Afterward, a second-by-second sampling was applied [45] resulting in one HR value for each of 6 s following stimulus onset (the whole stimulus duration). The HR in the last second prior to stimulus onset represented the prestimulus baseline and was subtracted from all HR values during stimulus presentation. Finally, the average of all these ΔHR values was calculated. The data sets of three DPD patients had to be discarded due to very low quality of the ECG recording. Thus, HR analyses were based on n=19 DPD patients and n=15 patient controls.

### Statistical analyses

T-tests were used to assess differences between DPD patients and patient controls in tonic physiological activity during the 5 min rest period. Ratings of valence, arousal and vividness were compared between patient groups using t-tests (for neutral sounds) or 2×2×2 analyses of variance (ANOVAs) with patient group as between-subject factor and valence category (positive vs. negative) and arousal category (medium vs. high arousal) as within-subject factors, respectively. To assess discrepancies between affective ratings and IADS norm ratings, we calculated differences between each participant’s ratings and gender-specific norm ratings. These differences were compared to 0 as well as between groups using a one-way ANOVA (for neutral sounds) with patient group as between-subject factor or a 2×2×2 ANOVAs (for emotional sounds) with the additional within-subject factors valence category (positive vs. negative) and arousal category (medium vs. high arousal). Ratings of mindful breathing effects were subjected to a 2×2 ANOVA using patient group as between-subject factor and experimental condition (normal listening vs. mindful breathing) as within-subject factor. Finally, SCR amplitudes and HR changes that were elicited by the neutral sounds were analyzed using 2×2 ANOVAs with patient group as between-subject factor and experimental condition (normal listening vs. mindful breathing) as within-subject factor; corresponding responses to the emotional sounds were analyzed using 2×2×2× 2 ANOVAs with the additional within-subject factors valence category (positive vs. negative) and arousal category (medium vs. high arousal). Significant interaction effects in the ANOVAs were followed by post-hoc t-tests with Bonferroni correction. Finally, we correlated affective ratings and physiological responses with anxiety (STAI-T/-S) and depression scores (BDI-II) using Pearson’s product-moment correlation coefficient.

An a priori significance threshold of α=.05 was used for all analyses. Partial η^2^ (for main and interaction effects in the ANOVAs) or Cohens’s d (for comparisons of two groups) are reported as effect size estimates.

## Results

### Psychometric data of DPD and control patients

There were no differences between both groups for age, sex, years of education, severity of current depression, anxiety, symptoms of dissociative amnesia and childhood traumatic experiences. However, severity of depersonalization and self-rated mindfulness differed strongly between the groups (see Table **1**).

### Tonic physiological activity

As shown in Table 2, physiological activity during the 5 min rest period was very similar between DPD patients and patient controls. A significant difference between groups was only observed for the number of NSRs with DPD patients showing more NSRs than patient controls.

**Table 2:** Electrodermal and heart rate data during a rest period of 5 minutes.

	DPD	Patient controls	t-Test
	n=22	n=15	p
SCL (log[μS])	6.3 ± 5.0	4.4 ± 4.4	0.22
#NSR	29.6 ± 25.7	13.1 ± 17.2	0.03
Heart rate (bpm)^1^	78.7 ± 10.2	78.4 ± 11.2	0.94
SDNN^1^	39.3 ± 13.5	53.5 ± 36.3	0.17
RMSSD^1^	25.1 ± 12.0	41.6 ± 36.9	0.11
HF (ms^2^)^1^	359.2 ± 421.0	1264.6 ± 1909.8	0.09
HF (n.u.)^1^	38.3 ± 21.5	41.2 ± 24.6	0.71
LF/HF^1^	3.0 ± 3.0	2.4 ± 1.9	0.49

Note: SCL, Skin conductance level; #NSR, Number of non-specific electrodermal responses; SDNN standard deviation of NN intervals; RMSSD, Square root of the mean squared differences of successive NN intervals; HF, high frequency activity; LF, low frequency activity

1) Heart rate measures were analyzed for n=21 DPD patients and n=15 patient controls

### Sound ratings

**Figure 1:**
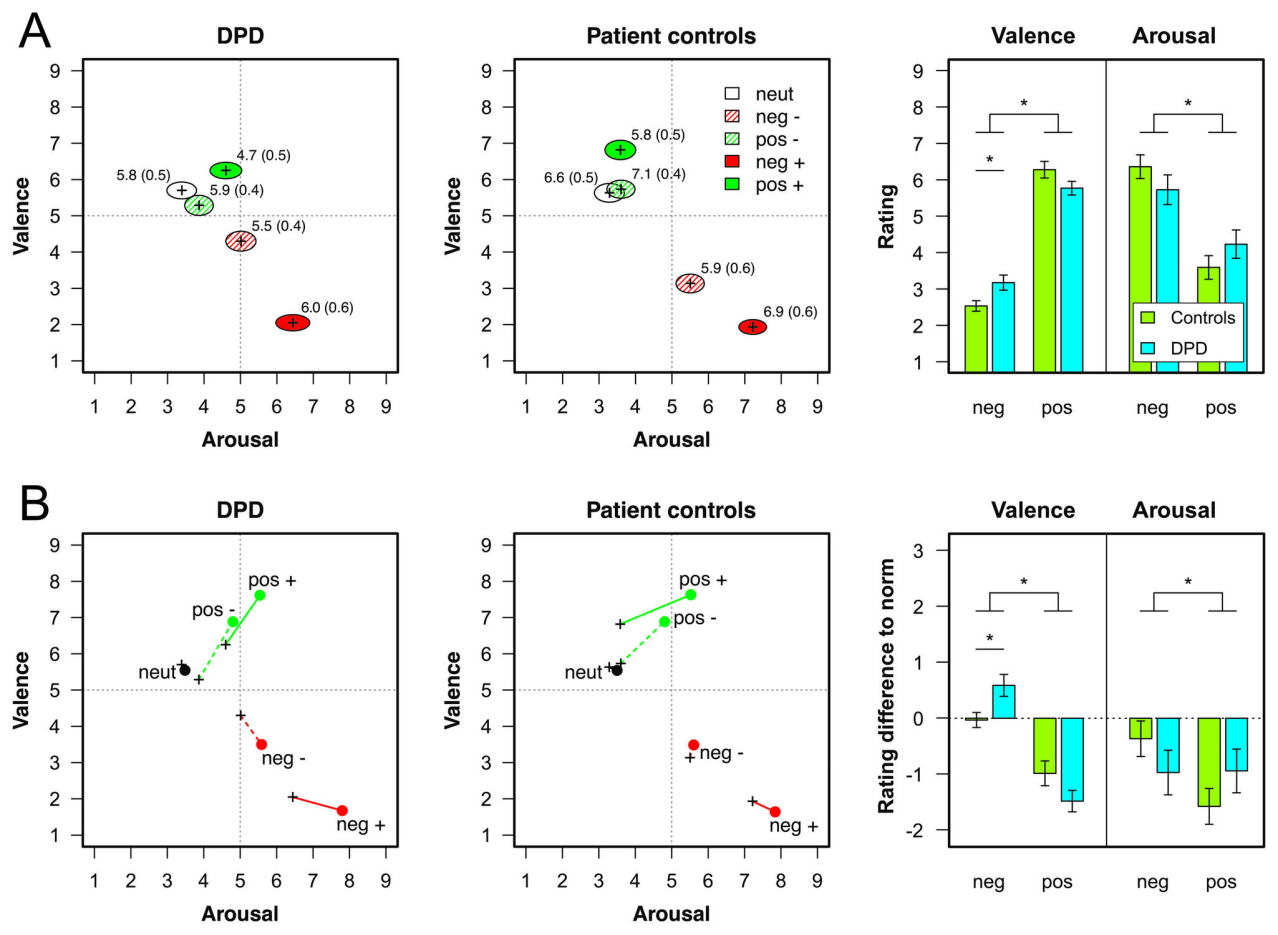
Valence and arousal ratings for neutral and emotional sounds. A) Valence and arousal ratings for neutral and emotional sounds as a function of group (DPD patients vs. patient controls). The center of the ellipses in the left and middle column represent the mean rating and the radii correspond to the standard error of mean (SEM). The values next to the ellipses depict mean and SEM for vividness ratings. The right column shows valence and arousal ratings collapsed across arousal categories as a function of group with error bars indicating SEM. B) Average differences to the norm ratings (Bradley & Lang, 2007) [30]. The dots in the left and middle column represent mean norm ratings and the crosses depict average ratings of each group. The bars in the right column depict differences to norm valence and arousal ratings collapsed across arousal categories as a function of group with error bars indicating SEM. neut = neutral, neg = negative, and pos = positive sounds; -/+ indicate medium and high arousal, respectively.

DPD patients and patient controls did not differ in their valence, t(33)<1, d=0.07, arousal, t(33)<1, d=0.06, or vividness ratings for neutral sounds, t(33)=1.21, p=.24, d=0.41. With respect to the emotional sounds, the ANOVA on the valence ratings revealed a significant main effect of valence category, F(1,33)=132.53, p<.001, η^2^=.80, indicating that patients of both groups rated positive sounds as pleasant and negative ones as unpleasant. Moreover, as revealed by a significant valence × arousal category interaction, F(1,33)=20.44, p<.001, η^2^=.38, this difference was more pronounced for highly arousing, t(34)=17.58, p<.001, d=3.00, as compared to medium arousing sounds, t(34)=5.37, p<.001, d=0.91. Finally, a significant group × valence category interaction was obtained, F(1,33)=7.13, p<.05, η^2^=.18. DPD patients rated unpleasant sounds as less unpleasant than patient controls, t(33)=2.36, p<.05, d=0.81, whereas no such difference was observed for positive sounds, t(33)=1.72, p=.19, d=0.59 (see Figure 1A, right panel).

The ANOVA on the arousal ratings of emotional sounds revealed significant main effects of arousal category, F(1,33)=7.85, p<.01, η^2^=.19, and valence category, F(1,33)=67.23, p<.001, η^2^=.67, indicating that highly arousing as well as negative sounds were rated more arousing. Furthermore, similar to the valence ratings, a significant interaction of valence × arousal category was obtained, F(1,33)=11.51, p<.01, η^2^=.26. Thus, arousal ratings differed significantly between medium and highly arousing negative sounds, t(34)=6.10, p<.001, d=1.03, whereas no such difference was obtained for positive sounds, t(34)=1.55, p=.26, d=0.26. Finally, a significant group × valence category interaction, F(1,33)=8.13, p<.01, η^2^=.20, indicates that differences in arousal ratings between negative and positive sounds varied between groups. However, corresponding post-hoc tests within each valence category did not reveal significant differences between groups.

Finally, the ANOVA on the vividness ratings only revealed a significant valence × arousal category interaction, F(1,33)=9.44, p<.01, η^2^=.22. Across groups, vividness ratings only differed between medium and highly arousing positive sounds, t(34)=4.62, p<.001, d=0.78, but not for negative sounds, t(34)=1.75, p=.18, d=0.30 (see values in Figure 1A).

With respect to the comparison to IADS norm ratings of neutral sounds, we did not observe significant effects for the intercept or the patient group, neither for valence, nor for arousal ratings. Thus, affective ratings of neutral sounds were very similar to the norm sample and did not differ between patient groups (Figure 1B)

With respect to differences in valence ratings of emotional sounds, we obtained a significant intercept, F(1,33)=13.64, p<.001, η^2^=.29, significant main effects of valence category, F(1,33)=9.33, p<.01, η^2^=.22, and arousal category, F(1,33)=4.81, p<.05, η^2^=.13, as well as a significant interaction of patient group × valence category, F(1,33)=7.31, p<.05, η^2^=.18. As can be seen from Figure 1B, valence ratings differed substantially from IADS norm ratings. This difference was larger for positive than for negative and to medium arousing as compared to highly arousing sounds. Whereas the valence of negative sounds was rated relatively similar between patient controls and the norm sample, t(33)=1.70, p=.20, d=0.58, these sounds were rated significantly more neutral in DPD patients, t(33)=2.42, p<.05, d=0.83.

A comparable analysis on the differences in arousal ratings of emotional sounds revealed a significant intercept, F(1,33)=6.95, p<.05, η^2^=.17, as well as significant main effects of valence, F(1,33)=13.41, p<.001, η^2^=.29, and arousal category, F(1,33)=4.25, p<.05, η^2^=.11. Overall, arousal ratings were lower than in the norm sample (Figure 1B). Moreover, larger discrepancies were observed for positive as compared to negative sounds as well as for highly arousing as contrasted with medium arousing sounds. The ANOVA additionally yielded a significant group × valence category interaction, F(1,33)=8.04, p<.01, η^2^=.20, indicating that differences in arousal ratings for negative sounds were more pronounced for DPD patients (Figure 1B, right panel). However, post-hoc tests within each valence category did not reveal significant differences between groups.

### Ratings of mindful breathing effects

**Figure 2:**
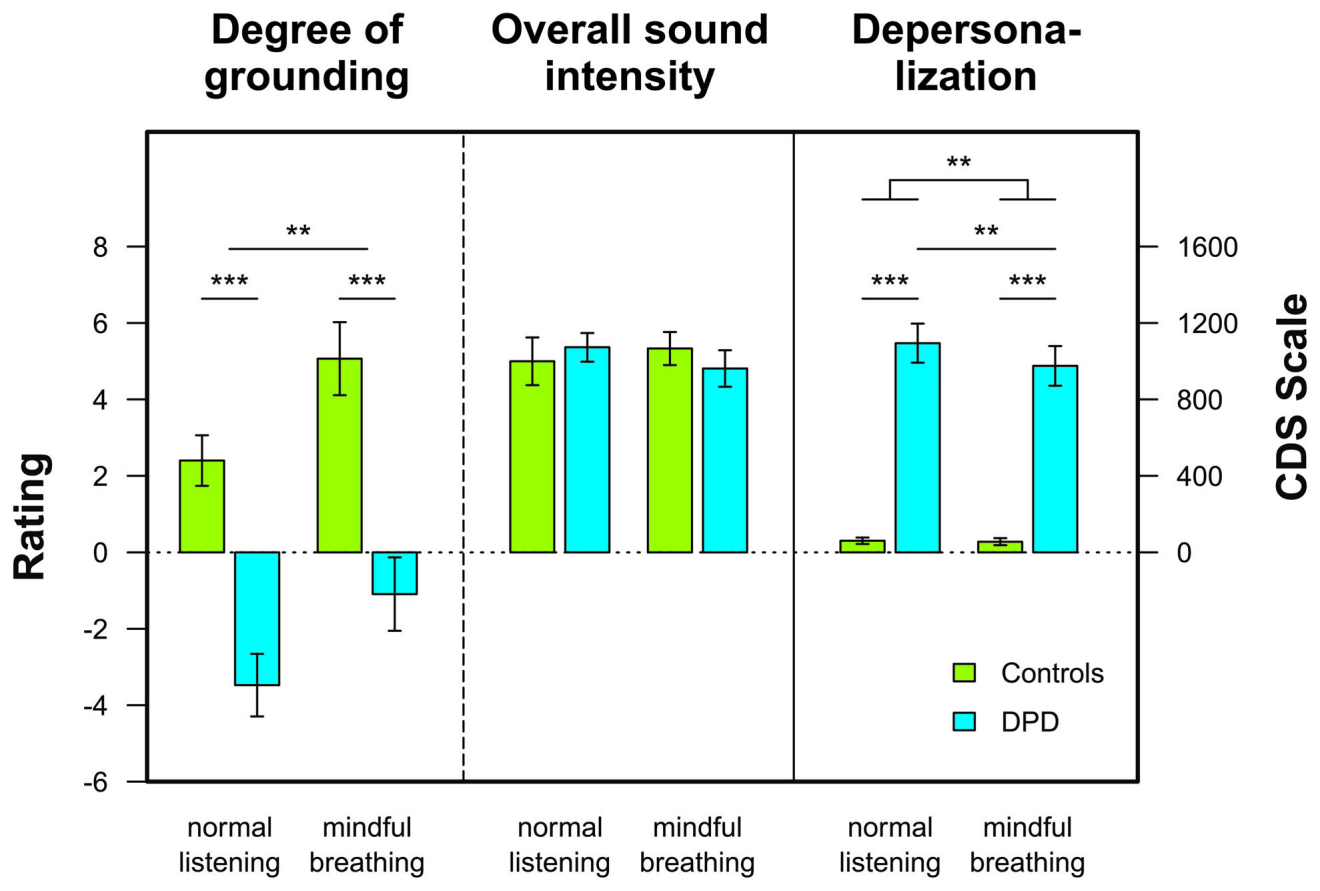
Effects of mindful breathing. Average ratings of the degree of grounding, the overall sound intensity, and results of the state version of the Cambridge Depersonalization Scale (S-CDS) as a function of group (DPD patients vs. patient controls) and mindfulness manipulation (normal listening vs. mindful breathing). Error bars indicate SEM.

The mindful breathing manipulation enhanced the degree of feeling grounded in both groups, F(1,33)=8.85, p<.01, η^2^=.21. DPD patients felt significantly less grounded than patient controls in both conditions, F(1,33)=32.14, p<.001, η^2^=.49 (see Figure 2). State depersonalization as assessed by the S-CDS was higher in DPD patients than in patient controls, F(1,34)=61.40, p<.001, η^2^=.64. As indicated by a significant group × experimental condition interaction, F(1,34)=5.61, p<.01, η^2^=.14, mindful breathing significantly reduced DP intensity in the DPD group, t(20)=2.99, p<.01, d=0.65, but not in patient controls, t(14)<1, d=0.19 (see Figure 2). With respect to the subjective intensity of the sounds, no significant effect was obtained in the ANOVA. Thus, ratings did not differ between groups and the mindful breathing manipulation did not modulate self-rated intensity ratings.

### Physiological responses to emotional sounds

**Figure 3:**
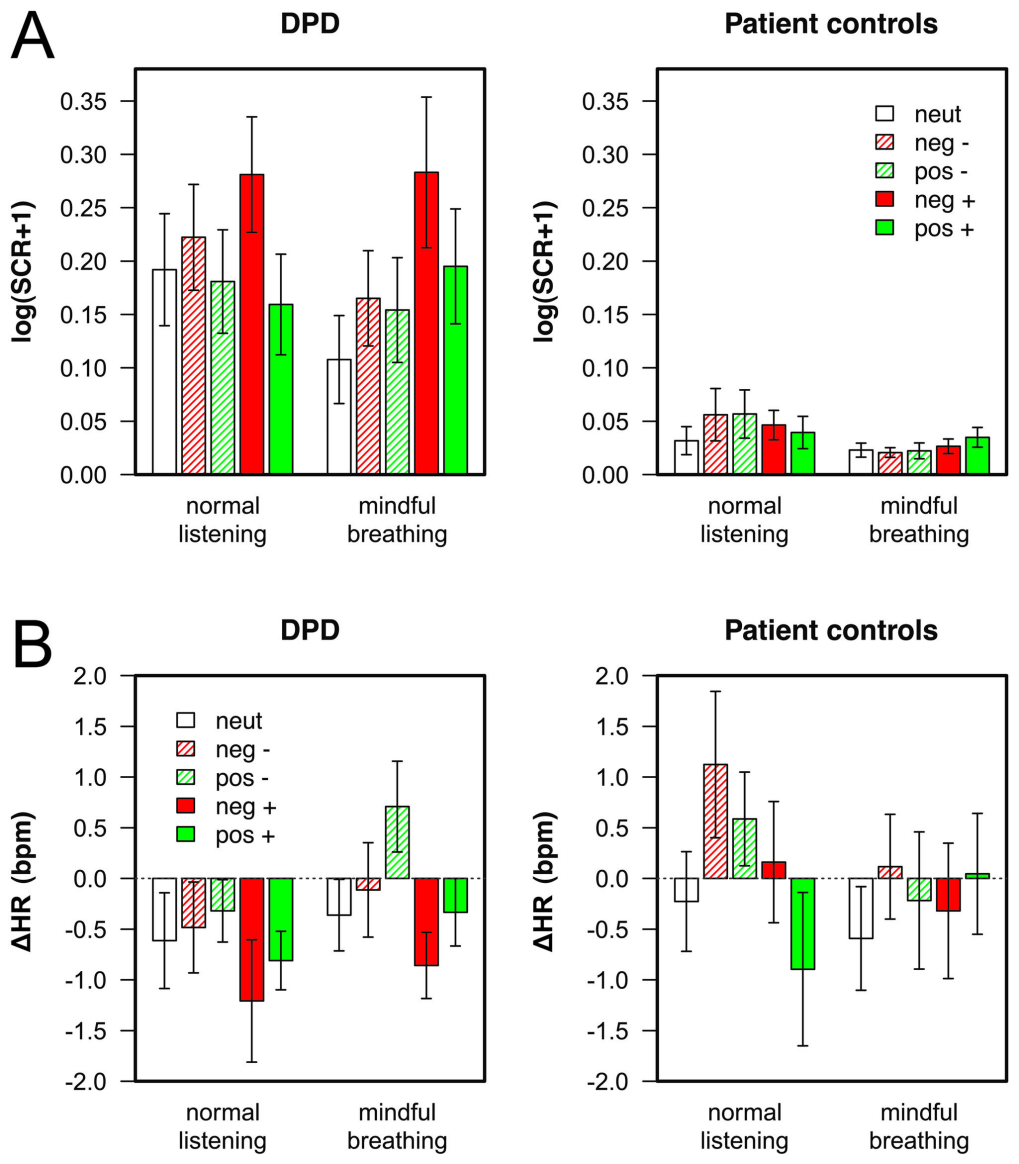
Physiological responses elicited by neutral and emotional sounds. Physiological responses elicited by neutral and emotional sounds as a function of group (DPD patients vs. patient controls) and mindfulness manipulation (normal listening vs. mindful breathing). A) Skin conductance response (SCR) amplitudes, B) phasic heart rate (HR) responses. Error bars indicate SEM, neut = neutral, neg = negative, and pos = positive sounds; -/+ indicate medium and high arousal, respectively.

**Figure 4:**
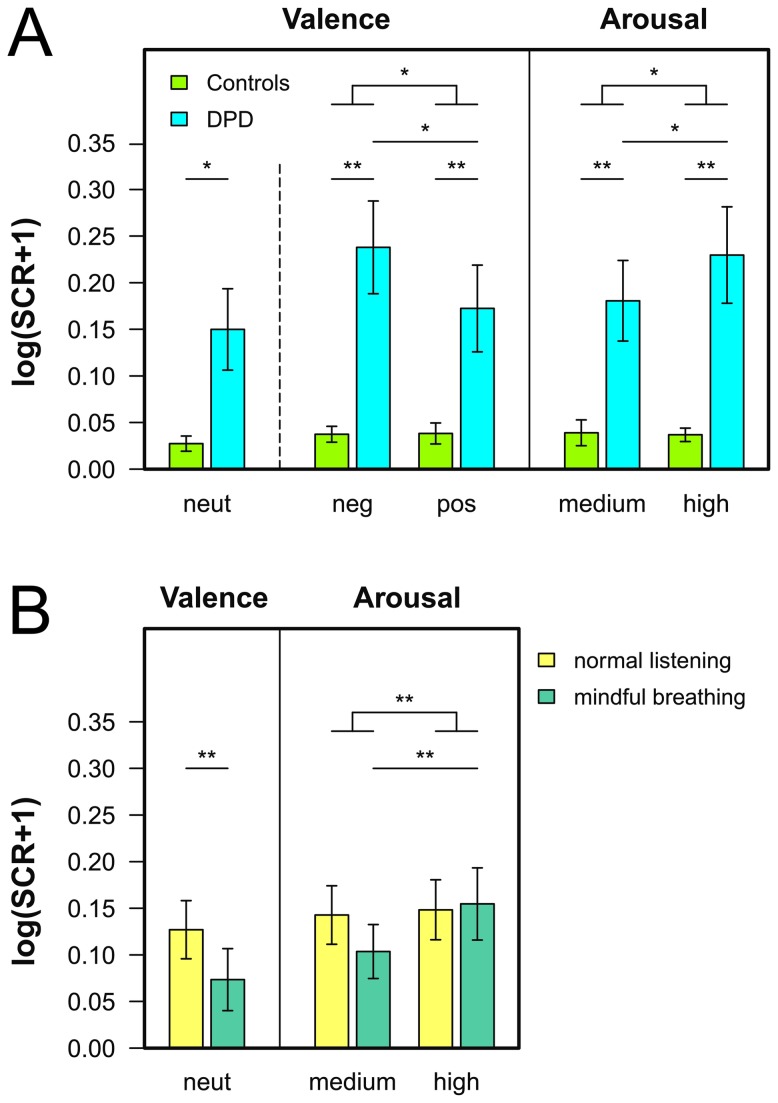
Illustration of significant effects in the electrodermal responses. A) Average skin conductance responses aggregated across valence or arousal categories as a function of group (DPD patients vs. patient controls). B) Average electrodermal responses aggregated across arousal categories as a function of the mindfulness manipulation (normal listening vs. mindful breathing). Error bars indicate SEM, neut = neutral, neg = negative, and pos = positive sounds.

The ANOVA on the SCR amplitudes for neutral sounds yielded significant main effects of the mindful breathing manipulation, F(1,35)=8.82, p<.01, η^2^=.20, and the patient group, F(1,35)=5.22, p<.05, η^2^=.13. Thus, SCR amplitudes were higher for DPD patients and decreased under mindful breathing in both groups (see Figures 3A and 4). The ANOVA for emotional sounds revealed significant main effects of group, F(1,35)=8.44, p<.01, η^2^=.19, valence category, F(1,35)=14.92, p<.001, η^2^=.30, and arousal category, F(1,35)=11.19, p<.01, η^2^=.24. However, these main effects were qualified by significant interactions of group × valence category, F(1,35)=6.21, p<.05, η^2^=.15, group × arousal category, F(1,35)=4.93, p<.05, η^2^=.12, and arousal × valence category, F(1,35)=10.02, p<.01, η^2^=.22. As shown in Figure 4A, DPD patients responded more strongly to the emotional sounds than patients controls. Furthermore, DPD patients showed larger SCR amplitudes to negative as compared to positive sounds, t(21)=3.05, p<.05, d=0.65, and to highly as compared to medium arousing sounds, t(21)=2.84, p<.05, d=0.61. Such differential response was not observed for patient controls, neither for a valence modulation, t(14)<1, d=0.04, nor an arousal modulation, t(14)<1, d=0.05. Across groups, SCR amplitudes were larger for highly as compared to medium arousing negative sounds, t(36)=2.82, p<.05, d=0.64, whereas no such difference was observed for positive sounds, t(36)<1, d=0.07.

Finally, we obtained a significant mindful breathing × arousal category interaction, F(1,35)=8.95, p<.01, η^2^=.20, indicating that highly arousing sounds elicited larger SCR amplitudes than medium arousing sounds in the mindful breathing condition, t(36)=3.22, p<.01, d=0.53, whereas no such global effect was observed under normal listening conditions, t(36)<1, d=0.07 (see Figure 4B).

In the ANOVA on the heart rate responses elicited by neutral sounds, no significant effect was obtained. Thus, HR responses were comparable between groups and unaffected by the mindful breathing manipulation. In the ANOVA on emotional sounds, only a significant main effect of arousal condition was obtained, F(1,32)=4.51, p<.05, η^2^=.12, indicating that highly arousing sounds were more likely to elicit a HR deceleration than medium arousing sounds (Figure 3B).

Correlation analyses did not reveal significant associations of anxiety or depression scores with electrodermal or heart rate responses, neither in the whole sample nor in the two groups.

## Discussion

The main findings were: DPD patients and patient controls, matched for severity of depression and anxiety, showed similar low levels of traumatic childhood experiences and of dissociative amnesia. However, dispositional mindfulness was much lower for DPD patients (d=0.92). DPD patients showed more non-specific skin conductance responses than patient controls, but had similar skin conductance levels, HR and HRV during the rest period. DPD patients rated unpleasant sounds as less unpleasant (i.e. more neutral) as compared to patient controls (d=0.81) and to IADS norm ratings (d=0.83). Comparable differences were also observed when comparing both patient groups to healthy individuals (see supplemental online material). Despite their neutralizing ratings, DPD patients showed overall stronger electrodermal responses to emotional sounds than patient controls and their SCR amplitudes depended on emotional valence and arousal whereas no such modulation was observed for the control group. These differences were also evident when comparing the response pattern of both patient groups to healthy individuals (see supplemental online material). Both patient groups experienced an increase of feeling grounded by mindful breathing, but only DPD patients endorsed a significant reduction of DP intensity (d=0.65). Across both groups, mindful breathing enhanced differential electrodermal responses to highly and medium arousing sounds. Neither anxiety nor depression had an effect on autonomic responses, neither in the whole sample, nor in the two groups.

Contrary to our expectations and to previous studies [17,18], showing attenuated autonomic responsiveness to negative emotional stimuli, we found increased electrodermal responsiveness for DPD patients as compared to patient controls. The amplitudes of skin conductance responses to negative sounds of the DPD patients were more similar to those of healthy controls. In this regard, it seems very important to note, that Sierra et al. [17] failed to observe significant differences in electrodermal responsiveness between DPD patients and healthy controls. Thus, despite high depersonalization and anxiety scores, their autonomic responses were more similar to those of healthy persons than to those of patients with anxiety disorders [17]. This may challenge the prevailing view of a blunted autonomic responsiveness in DPD. In our study, the patient controls showed overall less strong electrodermal responses and no modulation by valence and arousal. This pattern, which is in line with previous studies on emotional reactivity in major depressive disorder [46], may reflect a generally reduced emotional reactivity such as anhedonia [47]. In accordance with Schoenberg et al. (2012) [21], DPD patients showed more NSRs during a rest period, which reflects higher sympathetic lability [48]. Interestingly, electrodermal lability is associated with increased alertness, effortful control of emotional impulses [49] and introversion [48], each characteristic for DPD [9,12,50].

Did sample differences contribute to the discrepant findings? The psychometric characteristics of our DPD sample match with severe and chronic cases of DPD [51,52]. They had markedly higher scores in trait CDS and BDI than reported in previous psychophysiological studies [18,20,21] but anxiety scores were only slightly higher than in Sierra et al. (2006) [17]. Of note, only patients with anxiety disorders were examined as controls in this previous study, while in the current experiment the group of the patient controls was more heterogeneous and showed a high comorbidity with depression. The scores for BDI and STAI, however, differed only slightly between studies. It seems unlikely that the heterogeneity of comorbid conditions confounded our results, because anxiety and depression scores were well balanced between the two groups. Heterogeneity of comorbid conditions should not constitute an important drawback, as this study aimed to evaluate the role of depersonalization for emotion processing irrespective of comorbidities. The overall slightly higher psychometric scores of our sample may be due to the inpatient status of most participants. In sum, it seems unlikely that our discrepant findings are due to sample characteristics.

Concerning effects of mindful breathing, we showed for the first time that mindful breathing immediately reduced state depersonalization in DPD patients (d=0.65), increased feelings of being grounded and enhanced differential electrodermal responses to highly and medium arousing sounds. We assume that directing the attention to the physical sensations of breathing while listening to the affective sounds, increased the affective-sensory processing of the emotional sounds as reflected in the improved electrodermal modulation of the emotional stimuli. As a result, DPD patients became more grounded by attending to their breathing instead of losing themselves in ruminative self-observation as reflected in a momentary decrease of depersonalization severity. Cognitive-behavioral and psychodynamic conceptualizations consider ruminative and detached self-observation as a core mechanism contributing to the symptom building and maintenance of depersonalization [12,53,54]. Mindfulness exercises are supposed to direct attentional resources to limbic and insular cortices [25] pathways that—in DPD—are proposed to be suppressed by fronto-limbic inhibition, presumably mediated via attentional mechanisms [13]. Thus mindfulness may strengthen insular processing of emotional stimuli and thus helps overcoming impaired self-awareness.

By interpreting the results of our study, the following limitations seem crucial: We cannot preclude, that discrepant findings may be due to the different modality of emotional stimulation (auditory vs. visual). However, it has been shown in normative samples that acoustic cues yield similar affective responses as compared to pictures (see also supplement, Text S3), albeit acoustic stimulation may be associated with less modulated responses [55]. Another putative confound constitutes the instruction of keeping eyes closed during stimulus presentation. A recent neuroimaging study showed that closing the eyes while listening to emotional music, resulted in enhanced ratings of emotionality and greater activation of the amygdala (as well as Locus Coeruleus and Ventral Prefrontal Cortex) [56]. Moreover, as compared to healthy controls and patients with obsessive compulsive disorders, DPD patients showed reduced neural activation for affective visual stimuli in regions involved in visual processing (middle and superior temporal gyri), which might reflect lower attention to aversive stimuli [19]. It may be speculated, that dynamically evolving acoustic stimuli are less susceptible to suppressive mechanisms by attentional manipulations: You can more easily avert your eyes than shut your ears [55]. The lack of a matched healthy control group is limiting our conclusions concerning the comparison with healthy persons. However, comparison with healthy persons was not the primary aim of our study. Moreover, we could exploratively relate the present findings to a German sample of healthy individuals (see supplement, Text S3) and the normative sound ratings of a large healthy representative norm group [30].

In sum, the present findings do not support the theory that early stages of emotional processing are impaired in DPD. DPD patients responded more strongly to emotional scenarios than patient controls and showed a more differentiated response pattern. Thus, autonomic responses of DPD patients were more similar to healthy persons [55]. However, they showed an aberrant evaluation of negative auditory stimuli, in such a manner that they “neutralized” actually unpleasant auditory scenarios. Thus, their cognitive evaluation seems to be disconnected from their bodily or autonomic responses, respectively. This may be in line with the observation, that DPD patients have greater difficulties in identifying own feelings as compared to other patients or healthy controls [57]. It also reminds of findings of hypoactive insula in DPD, as the insula is involved in the conscious representation of autonomic states of the body [13,17,58].

In conclusion, our findings have important implications for psychotherapeutic approaches: 1) Mindfulness exercises, which immediately decrease DP intensity, may be helpful by strengthening the modulation of autonomic responses and thus increasing self-awareness. 2) the DPD patient needs special help to increase his conscious awareness for bodily signals, and to recognize and evaluate his affective reactions properly, or to reword Paul Schilder (1939) “to acknowledge himself as a personality” [59].

This study extends evidence that DPD is associated with a specific impairment of emotion processing and thus constitutes a nosological entity in its own right. Further studies are needed to understand the complex interplay of affect, cognition and attention in DPD.

## Supporting Information

Table S1 **Current mental disorders other than DPD in both groups.**
(DOC)

Figure S1 **Valence and arousal ratings for neutral and emotional sounds.** The left panel shows mean ratings (center of ellipses) and SEM (radii of ellipses) for the current sample. The right panel depicts average differences to the norm ratings (Bradley & Lang 2007). The dots represent mean norm ratings and the crosses depict average ratings of the current sample. Neut = neutral, neg = negative, and pos = positive sounds; -/+ indicate medium and high arousal, respectively.
(TIF)

Figure S2 **Physiological responses elicited by neutral and emotional sounds.** Skin conductance response (SCR) amplitudes are depicted in the left panel and phasic heart rate (HR) responses in the right panel. Error bars indicate SEM, neut = neutral, neg = negative, and pos = positive sounds; -/+ indicate medium and high arousal, respectively.
(TIF)

Figure S3 **Differences in ratings (A) and electrodermal responses (B) between healthy controls, patients controls, and patients with depersonalization disorder (DPD).** Error bars indicate SEM, neut = neutral, neg = negative, and pos = positive sounds.
(TIF)

Text S1 **Additional information about the psychometric questionnaires.**
(DOCX)

Text S2 **Evaluation of the selected emotional sounds.**
(DOC)

Text S3 **Comparison of patient groups to healthy participants.**
(DOC)
