# Supplementary material for: Depersonalization Disorder: Disconnection of Cognitive Evaluation from Autonomic Responses to Emotional Stimuli
Source: PLoS One. 2013 Sep 13;8(9):e74331. doi: 10.1371/journal.pone.0074331 (PMC3772934; doi:10.1371/journal.pone.0074331)
Supplement: Table S1 — Current mental disorders other than DPD in both groups. (DOC) [file pone.0074331.s001.doc]

**Table S1**

**Current mental disorders other than DPD in both groups**

|  | DPD | Patient controls |
| --- | --- | --- |
|  | n = 22 | n = 15 |
| **Axis I Disorders** | | |
| Major depression | 52.0% (13) | 60.0% (9) |
| Dysthymia | 44.0% (11) | 6.7% (1) |
| GAD | 16.0% (4) | 6.7% (1) |
| Social phobia | 16.0% (4) | 26.7% (4) |
| Agoraphobia/panic disorder | 20.0% (5) | 40.0% (6) |
| Specific phobia | 8.0% (2) | 6.7% (1) |
| Anxiety disorder NOS | 8.0% (2) | - |
| Obsessive compulsive disorder | 12.0% (3) | 20.0% (3) |
| Posttraumatic stress disorder | - | 6.7% (1) |
| Neurasthenia* | - | 6.7% (1) |
| Bulimia nervosa | - | 6.7% (1) |
| **Personality disorders (PD)** | | |
| Any PD | 56.0% (14) | 53.3% (8) |
| NOS PD | 24.0% (6) | 6.7% (1) |
| Anxious-avoidant PD | 16.0% (4) | 33.3% (5) |
| Dependent PD | 4.0% (1) | 6.7% (1) |
| Borderline PD | 8.0% (2) | 6.7% (1) |
| Obsessive-compulsive PD | 4.0% (1) | - |

*Note*: Data are presented as percentage and number (n)
DPD = depersonalization disorder; NOS = not otherwise specified; *Neurasthenia according to ICD-10: F48.0, neurasthenia is characterized by distressing feelings of exhaustion, muscular aches; dizziness; tension headache; sleep disturbance; inability to relax, neurasthenia is not included in the DSM-IV

Current psychotropic medication: In the DPD group 12 patients were taking selective serotonin reuptake inhibitors (SSRIs), 3 patients additionally low dose atypical antipsychotics, and 1 person additionally lamotrigine. In the group of the patient controls 3 persons were taking SSRIs, 1 patient opipramol and 1 patient mirtazapine.
