# Supplementary material for: Depersonalization Disorder: Disconnection of Cognitive Evaluation from Autonomic Responses to Emotional Stimuli
Source: PLoS One. 2013 Sep 13;8(9):e74331. doi: 10.1371/journal.pone.0074331 (PMC3772934; doi:10.1371/journal.pone.0074331)
Supplement: Text S1 — Additional information about the psychometric questionnaires. (DOCX) [file pone.0074331.s005.docx]

## **Text S1**

## **Additional information about the psychometric questionnaires**

The selection of the questionnaires was based on the following considerations: Firstly, in order to enable the comparison of our sample with those of other DPD studies, we selected questionnaires, which were used in the majority of previous DPD studies [[1-13](#_ENREF_1)]. This applied to the Cambridge Depersonalization Scale (CDS) [[14](#_ENREF_14),[15](#_ENREF_15)], the Dissociative Experiences Scale (DES) and its subscales amnesia and depersonalization [[16](#_ENREF_16)], the Beck Depression Inventory-II (BDI-II) [[17](#_ENREF_17)] and the State-Trait Anxiety Inventory (STAI-T/-S) [[18](#_ENREF_18)]. Further, the BDI-II and the STAI were used to describe the impairment by symptoms of anxiety and depression, as there is a high comorbidity with depressive disorders and anxiety disorders in DPD. In the end, assessment of depression and anxiety should allow the matching of the DPD group with the patient controls. Secondly, the Childhood Trauma Questionnaire (CTQ) [[19](#_ENREF_19)] was included in order to describe the severity of traumatic childhood experiences. According to the DPD literature, DPD is not associated with severe forms of childhood trauma (e.g. sexual abuse) in contrast to severe dissociative disorders such as dissociative identity disorder [[13](#_ENREF_13)]. The CTQ enabled us to demonstrate, that the level of childhood traumatic experiences was not high in both groups. Thirdly, as we used a mindful breathing condition in our study, we applied the Mindful Attention Awareness Scale (MAAS) [[20](#_ENREF_20)] to measure dispositional mindfulness and to compare DPD patients with patient controls regarding their dispositional mindfulness.

**References of Text S1**

1. Schoenberg PL, Sierra M, David AS (2012) Psychophysiological investigations in depersonalization disorder and effects of electrodermal biofeedback. J Trauma Dissociation 13: 311-329.

2. Sierra M, Medford N, Wyatt G, David AS (2012) Depersonalization disorder and anxiety: a special relationship? Psychiatry Res 197: 123-127.

3. Mantovani A, Simeon D, Urban N, Bulow P, Allart A, et al. (2011) Temporo-parietal junction stimulation in the treatment of depersonalization disorder. Psychiatry Res 186: 138-140.

4. Simeon D, Giesbrecht T, Knutelska M, Smith RJ, Smith LM (2009) Alexithymia, absorption, and cognitive failures in depersonalization disorder: a comparison to posttraumatic stress disorder and healthy volunteers. J Nerv Ment Dis 197: 492-498.

5. Giesbrecht T, Merckelbach H, van Oorsouw K, Simeon D (2010) Skin conductance and memory fragmentation after exposure to an emotional film clip in depersonalization disorder. Psychiatry Res 177: 342-349.

6. Simeon D, Kozin DS, Segal K, Lerch B (2009) Is depersonalization disorder initiated by illicit drug use any different? A survey of 394 adults. J Clin Psychiatry 70: 1358-1364.

7. Guralnik O, Giesbrecht T, Knutelska M, Sirroff B, Simeon D (2007) Cognitive functioning in depersonalization disorder. J Nerv Ment Dis 195: 983-988.

8. Simeon D, Knutelska M, Nelson D, Guralnik O (2003) Feeling unreal: a depersonalization disorder update of 117 cases. J Clin Psychiatry 64: 990-997.

9. Simeon D, Guralnik O, Hazlett EA, Spiegel-Cohen J, Hollander E, et al. (2000) Feeling unreal: a PET study of depersonalization disorder. Am J Psychiatry 157: 1782-1788.

10. Medford N, Baker D, Hunter E, Sierra M, Lawrence E, et al. (2003) Chronic depersonalization following illicit drug use: a controlled analysis of 40 cases. Addiction 98: 1731-1736.

11. Sierra M, Senior C, Phillips ML, David AS (2006) Autonomic response in the perception of disgust and happiness in depersonalization disorder. Psychiatry Res 145: 225-231.

12. Sierra M, Baker D, Medford N, Lawrence E, Patel M, et al. (2006) Lamotrigine as an add-on treatment for depersonalization disorder: a retrospective study of 32 cases. Clin Neuropharmacol 29: 253-258.

13. Simeon D, Guralnik O, Schmeidler J, Sirof B, Knutelska M (2001) The role of childhood interpersonal trauma in depersonalization disorder. Am J Psychiatry 158: 1027-1033.

14. Sierra M, Berrios GE (2000) The Cambridge Depersonalization Scale: a new instrument for the measurement of depersonalization. Psychiatry Res 93: 153-164.

15. Michal M, Sann U, Niebecker M, Lazanowsky C, Kernhof K, et al. (2004) Die Erfassung des Depersonalisations-Derealisations-Syndroms mit der Deutschen Version der Cambridge Depersonalisation Scale (CDS). Psychother Psychosom Med Psychol 54: 367-374.

16. Bernstein EM, Putnam FW (1986) Development, reliability, and validity of a dissociation scale. The Journal of Nervous and Mental Disease 174: 727-735.

17. Beck AT, Steer R, Brown GK (1996) Beck Depression Inventory (BDI-II). San Antonio (TX): Psychological Corporation.

18. Spielberger CD, Gorsuch RL, Lushene RE (1970) Manual for the state-trait anxiety inventory.

19. Bernstein DP, Fink L (1998) Childhood trauma questionnaire: A retrospective self-report: Manual: Psychological Corporation.

20. Brown KW, Ryan RM (2003) The benefits of being present: mindfulness and its role in psychological well-being. J Pers Soc Psychol 84: 822-848.
