# Supplementary material for: Depersonalization Disorder: Disconnection of Cognitive Evaluation from Autonomic Responses to Emotional Stimuli
Source: PLoS One. 2013 Sep 13;8(9):e74331. doi: 10.1371/journal.pone.0074331 (PMC3772934; doi:10.1371/journal.pone.0074331)
Supplement: Text S2 — Evaluation of the selected emotional sounds. (DOC) [file pone.0074331.s006.doc]

## **Text S2**

## **Evaluation of the selected emotional sounds**

**Materials and Methods**

The subset of emotional stimuli from the International Affective Digitized Sounds (IADS) that was used for the patient study was previously evaluated in a pilot study with healthy subjects (n=20; 10 women, 10 men; mean age=24.1 years, SD=4.7 years). Stimulus presentation was comparable to the main study but took place in an air-conditioned, sound-attenuated chamber with participants seated in a semi-reclining chair. Sounds were played to the participants using standard hi-fi amplifier (Technics Stereo Integrated Amplifier SU-Z400) and loudspeakers (Jamo 7084). Volume was once adjusted to be comfortable for all sounds before the experiment started and remained constant across all participants. Participants rated valence and arousal for each sound using the Self-Assessment Manikin . Vividness ratings were not obtained in this pilot study.

The Computerized Polygraph System (CPS, Stoelting Company) was used to record electrodermal data with a sampling rate of 60 Hz. Measurement was accomplished by a constant voltage system (0.5 V) using a bipolar recording with two Hellige Ag/AgCl electrodes (surface area=1 cm^2^) filled with 0.05 M NaCl electrolyte placed on the thenar and hypothenar surfaces of the participant’s non-dominant hand. An electrocardiogram (ECG) was measured using two Hellige Ag/AgCl electrodes filled with electrode paste and attached to the manubrium sterni and the left lower rib cage. The reference electrode was placed at the right lower rib cage. ECG-data were registered by the Varioport-device (Vitaport system, Becker Meditec) with a sampling rate of 512 Hz. Comparable to the main experiment, we determined the largest increase in skin conductance during a period from 1 to 8 s after stimulus onset as change in μS and log-transformed these amplitude values. Phasic heart rate was determined from the ECG recordings and averaged across the 6 s sound presentation (see main article for further details).

Ratings of valence and arousal as well as SCR amplitudes and HR changes were analyzed using 2 × 2 analyses of variance (ANOVAs) with valence category (positive vs. negative) and arousal category (medium vs. high arousal) as within-subject factors. Discrepancies between affective ratings and IADS norm ratings were evaluated by calculating differences between each participant’s ratings and IADS norm ratings for men and women, respectively. These differences were compared to 0 using a one-sample t-test (for neutral sounds) and by using 2 × 2 ANOVAs with valence category (positive vs. negative) and arousal category (medium vs. high arousal) as within-subject factors (for emotional sounds).

Significant interaction effects in the ANOVAs were followed by post-hoc t-tests with Bonferroni correction. An a priori significance threshold of α = .05 was used for all analyses. Partial η^2^ (for main and interaction effects in the ANOVAs) or Cohens’s d (for comparisons of two groups) are reported as effect size estimates.

**Results and Discussion**

As can be seen from Figure S1 (left panel), sounds categories were well separated in this pilot study (**Figure S1 in Text S2**). The ANOVA on the valence ratings revealed a significant main effect of valence category, F(1,19)=999.82, p<.001, η^2^=.98, as well as significant interaction of valence and arousal category, F(1,19)=179.62, p<.001, η^2^=.90. Thus, participants rated positive sounds as pleasant and negative ones as unpleasant. This difference was more pronounced for highly arousing, t(19)=8.49, p<.001, d=1.90, as compared to medium arousing sounds, t(19)=6.25, p<.001, d=1.40. For arousal ratings, significant main effects of valence category, F(1,19)=47.67, p<.001, η^2^=.72, and arousal category were obtained, F(1,19)=79.29, p<.001, η^2^=.81, as well as a significant interaction of both factors, F(1,19)=6.71, p<.05, η^2^=.26. Thus, arousal ratings were higher for negative as compared to positive and for highly as compared to medium arousing sounds. Moreover, differences in arousal ratings as a function of arousal category were more pronounced for negative, t(19)=6.96, p<.001, d=1.56, as compared to positive sounds, t(19)=4.59, p<.001, d=1.03 (**Figure S1 in Text S2**).

The comparison to the norm ratings of neutral sounds revealed significant differences for valence, t(19)=2.32, p<.05, d=0.52, as well as arousal ratings, t(19)=2.18, p<.05,
d = 0.49. Thus, neutral sounds were rated as more neutral and less arousing in this study as compared to the IADS norm ratings (see Figure S1, right panel). In the ANOVA on valence ratings, we obtained a significant intercept, F(1,19)=29.58, p<.001, η^2^=.61, as well as a significant main effect of arousal category, F(1,19)=15.57, p<.001, η^2^=.45. Thus, valence ratings were overall smaller than previously reported (Bradley & Lang 2007) and this reduction was more pronounced for medium arousing sounds. The ANOVA on arousal ratings only yielded a significant effect for the intercept, F(1,19)=9.28, p<.01, η^2^=.36, indicating overall reduced arousal ratings as compared to the norm ratings (**Figure S1 in Text S2**, right panel).

With respect to the physiological responses, the ANOVA on SCR amplitudes only revealed a significant main effect of arousal category, F(1,19)=7.38, p<.05, η^2^=.28, with highly arousing sounds eliciting larger SCRs than medium arousing sounds (**Figure S2 in Text S2**). In the ANOVA on the HR changes, we obtained significant main effects of valence category, F(1,19)=4.90, p<.05, η^2^=.20, as well as of arousal category, F(1,19)=6.39, p<.05, η^2^=.25. Thus, negative sounds were accompanied by a larger cardiac deceleration as compared to positive sounds that tended to elicit an acceleration. However, decelerative responses were more pronounced for stimuli with medium arousal (**Figure S2 in Text S2**).

These data indicate the current selection of sounds from the IADS could be reliably arranged into different valence and arousal categories. Although we observed significant differences to the norm ratings, this discrepancy mainly represents a global effect with reduced valence and arousal ratings for all sounds. Only with respect to the valence ratings, this effect was found to be less pronounced for highly arousing sounds. Taken together, the overall pattern of subjective ratings was very similar to the original norm sample .

With respect to the physiological responses, we replicated previously reported arousal effects on electrodermal responses . However, in contrast to this previous study, heart rate was modulated by valence across all sounds and not only for highly arousing sounds. The overall pattern of physiological responsiveness is comparable to visual stimulus material , thus rendering it highly useful for studying affective responses to acoustic stimuli in depersonalization disorder.

**References of Text S2**

1. Bradley MM, Lang PJ (2007) The International Affective Digitized Sounds (; IADS-2): Affective ratings of sounds and instruction manual. University of Florida, Gainesville, FL, Tech Rep B-3.

2. Bradley MM, Lang PJ (1994) Measuring emotion: the Self-Assessment Manikin and the Semantic Differential. J Behav Ther Exp Psychiatry 25: 49-59.

3. Bradley MM, Lang PJ (2000) Affective reactions to acoustic stimuli. Psychophysiology 37: 204-215.

4. Lang PJ, Greenwald MK, Bradley MM, Hamm AO (1993) Looking at pictures: affective, facial, visceral, and behavioral reactions. Psychophysiology 30: 261-273.

5. Bradley MM, Miccoli L, Escrig MA, Lang PJ (2008) The pupil as a measure of emotional arousal and autonomic activation. Psychophysiology 45: 602-607.

**Supporting Information Legends of Text S2**

**Figure S1. Valence and arousal ratings for neutral and emotional sounds.**

The left panel shows mean ratings (center of ellipses) and SEM (radii of ellipses) for the current sample. The right panel depicts average differences to the norm ratings (Bradley & Lang 2007). The dots represent mean norm ratings and the crosses depict average ratings of the current sample. Neut = neutral, neg = negative, and pos = positive sounds; -/+ indicate medium and high arousal, respectively.

**Figure S2. Physiological responses elicited by neutral and emotional sounds.**

Skin conductance response (SCR) amplitudes are depicted in the left panel and phasic heart rate (HR) responses in the right panel. Error bars indicate SEM, neut = neutral, neg = negative, and pos = positive sounds; -/+ indicate medium and high arousal, respectively.
