# Supplementary material for: Depersonalization Disorder: Disconnection of Cognitive Evaluation from Autonomic Responses to Emotional Stimuli
Source: PLoS One. 2013 Sep 13;8(9):e74331. doi: 10.1371/journal.pone.0074331 (PMC3772934; doi:10.1371/journal.pone.0074331)
Supplement: Text S3 — Comparison of patient groups to healthy participants. (DOC) [file pone.0074331.s007.doc]

**Text S3**

**Comparison of patient groups to healthy participants**

To directly compare whether the significant differences between patient controls and patients with depersonalization disorder (DPD) also generalize to comparisons with the above mentioned sample of healthy participants, we calculated several ANOVAs including the latter group of subjects. These analyses focused on the normal listening condition of the main study since this condition was most similar to the procedure in the study with healthy controls.

**Results and Discussion**

First, to examine differences in valence ratings as a function of valence category, we calculated a 3×2 ANOVA with group as between-subject factor and valence category (negative vs. positive) as within-subject factor. This analysis revealed a significant main effect of valence category, F(1,52)=36.07, p<.001, η^2^=.41, indicating that negative sounds were rated more negative than positive ones. However, similar to the analyses in the main article, we also obtained a significant group × valence category interaction, F(2,52)=5.30, p<.01, η^2^=.17. As depicted in Figure S3A, this interaction is mainly driven by DPD patients who rated the sounds as more neutral (i.e., closer to 5, the midpoint of the valence scale that represents “neutral”) (**Figure S3 in Text S3**).

Second, we compared differences in electrodermal responses to neutral sounds between all three groups. A oneway ANOVAs with group as between-subject factor yielded a significant effect, F(2,54)=4.03, p<.05, η^2^=.13. Figure S3B shows that DPD patients, but also healthy controls, responded stronger to neutral sounds than patient controls. To examine the modulation of electrodermal responses by valence and arousal, we carried out two 3×2 ANOVAs with group as between-subject factor and valence or arousal category, respectively, as within-subject factor. The ANOVA on the valence modulation yielded significant effects of group, F(2,54)=5.45, p<.01, η^2^=.17, and group × valence category, F(2,54)=4.12, p<.05, η^2^=.13. The ANOVA on the arousal modulation revealed significant effects of group, F(2,54)=3.52, p<.05, η^2^=.12, arousal category, F(1,54)=5.67, p<.01, η^2^=.10, and group × arousal category, F(2,54)=7.54, p<.01, η^2^=.22. As depicted in Figure S3B, DPD patients and healthy controls responded stronger to emotional sounds as compared to patient controls (**Figure S3 in Text S3**). Moreover, a modulation of the response pattern by valence and arousal was absent in the latter group whereas it was evident in DPD patients (more pronounced for valence category) and healthy controls (more pronounced for arousal category).

To sum up, these analyses indicate that DPD patients rated emotional sounds as more neutral even when compared to healthy individuals. Moreover, DPD patients showed larger electrodermal responses than both other groups but the modulation of skin conductance responses as a function of valence and arousal was more comparable between DPD patients and healthy controls. Patient controls failed to show such modulation. Although these analyses strengthen the conclusions of the main study, they have to be treated with caution since the experimental procedure and the equipment for measuring autonomic data differed between the sound evaluation study with healthy controls and the main patient study. Moreover, healthy individuals were not matched to patients with respect to age, sex, or years of education. Future studies explicitly addressing differences between healthy individuals and patient groups should take these issues into account.

**Supporting Information Legends of Text S3**

**Figure S3.** **Differences in ratings (A) and electrodermal responses (B) between healthy controls, patients controls, and patients with depersonalization disorder (DPD).**

Error bars indicate SEM, neut = neutral, neg = negative, and pos = positive sounds
